# Two Novel Short Peptidoglycan Recognition Proteins (PGRPs) From the Deep Sea Vesicomyidae Clam *Archivesica packardana*: Identification, Recombinant Expression and Bioactivity

**DOI:** 10.3389/fphys.2018.01476

**Published:** 2018-10-23

**Authors:** Xue Kong, Helu Liu, Yanan Li, Haibin Zhang

**Affiliations:** ^1^Institute of Deep-Sea Science and Engineering, Chinese Academy of Sciences, Sanya, China; ^2^College of Earth and Planetary Sciences, University of Chinese Academy of Sciences, Beijing, China

**Keywords:** innate immunity, *Archivesica packardana*, PGRP, antibacterial activity, endosymbiosis

## Abstract

Vesicomyidae clams are common species living in cold seeps, which incorporates symbiotic bacteria into their body maintaining endosymbiosis relationship. As members of pattern recognition receptor (PRR) family, peptidoglycan recognition proteins (PGRPs) recognize pathogen associated molecular patterns and play an important role in innate immunity. In present study, two short PGRPs (ApPGRP-1 and -2) were first identified from Vesicomyidae clam *Archivesica packardana*. Sequences analysis showed that they have both conserved Zn^2+^ binding sites (H-H-C) and amidase catalytic sites (H-Y-H-T-C), and phylogenetic tree indicated that they clustered with short PGRPs of other molluscs. PGN assay showed that ApPGRPs could bind Lys-type PGN from *Staphylococcus aureus* and Dap-type PGN from *Bacillus subtilis*, and revealed amidase activity with selective zinc ion dependence. rApPGRP-1 and -2 (recombinant ApPGRP-1 and -2) could bind six bacteria with a broad spectrum and had both zinc-dependent and -independent bactericidal activity. ApPGRPs had the complete functions of effectors and partial functions of receptors from PGRPs. Further analyses showed that ApPGRPs from *A. packardana* might be involved in the endosymbiosis relationship between the host clam and endosymbiotic bacteria as a regulator. The results of these experiments suggested that ApPGRPs were involved in cold seep clams’ immune response. This study provides basic information for further research on the immune mechanisms of deep sea organisms.

## Introduction

The immune system contains innate immunity and adaptive immunity (Janeway, 1989). Innate immune system is the frontline of defense and almost the only defense mechanism for invertebrates to protect the host from invasion by microbes in the surrounding environment (Janeway, 1989). Innate immunity can be triggered by a set of specific receptors termed PRRs. PRRs could recognize the conserved, invariant components on the cell surface of microbes named PAMPs (Yang et al., 2013). PRRs include Toll-like receptors, PGRP, scavenger receptor (SRCR), thioester-containing proteins (TEP), lipopolysaccharide and beta-1,3-glucan binding protein (LGBP), and lectins (Janeway and Medzhitov, 2002; Hoffmann, 2003).

As a subset of PRRs, PGRP recognizes peptidoglycan (PGN) which is a crucial cell wall component of microorganisms (Steiner, 2002). In PGN, *N*-acetylglucosamine (GlcNAc) and *N*-acetylmuramic acid (MurNAc) are linked by beta (1-4)- glycoside bond. MurNAc can be linked to short peptides that have three to five commutative L and D amino acids (Chen and Lv, 2014). Therefore, PGN could be divided into two types: L-lysine-type (Lys-type) and meso-diaminopimelic acid-type (Dap-type) according to the third residue of the short peptides (Yao et al., 2012). PGRPs were divided into three types: small extracellular PGRPs (PGRP-S, 20–25 KDa), long PGRPs (PGRP-L, >90 KDa), and intermediate PGRPs (PGRP-I, 40–45 KDa) (Ni et al., 2007).

Peptidoglycan recognition proteins are identified in multiple species, from insects to mammals (Dziarski, 2003; Dziarski and Gupta, 2006). Generally, their functions include three classes. Firstly, as receptors, PGRPs recognize microbes and then activate Toll or IMD pathways for AMP synthesis to remove invasive bacteria (Tzou, 2002; Dziarski, 2003). Secondly, as regulators, PGRPs act as negative factors to diminish or shut down IMD pathways to maintain the homeostasis of symbiotic bacteria (Guo et al., 2014). PGRPs can also enhance phagocytosis (Garver et al., 2006; Royet and Dziarski, 2007). Finally, as effectors, PGRPs can kill bacteria with amidase activity, which disrupt the lactylamide bond between muramic acid and L-alanine (Gelius et al., 2003; Kim et al., 2003; Chen et al., 2014).

Compared with those in insects and mammals, studies on marine organisms PGRPs were also carried out. In fish, researchers have shown that PGRPs from *Pseudosciaena crocea*, *Sciaenops ocellatus*, *Branchiostoma japonicum*, *Ctenopharyngodon idella*, *Ictalurus punctatus*, *Cynoglossus semilaevis*, *Oreochromis niloticus*, and *Scophthalmus maximus L.* were all constitutively expressed or up-regulated in response to pathogen or PAMP challenge (Yong et al., 2010; Li et al., 2012, 2014a,b; Yao et al., 2012; Sun et al., 2014; Sun and Sun, 2015; Gan et al., 2016; Zhang et al., 2016). In Echinodermata, PGRP-S2a from European sea star *Asterias rubens* breaks down peptidoglycan and stimulates phagocytosis of *Micrococcus luteus* through sea star phagocytes (Coteur et al., 2007). In molluscs, studies of PGRPs from *Argopecten irradians* (Ni et al., 2007), *Crassostrea gigas* (Itoh and Takahashi, 2009), *Solen grandis* (Wei et al., 2012), *Hyriopsis cumingi* (Yang et al., 2013), and *Haliotis discus discus* (Premachandra et al., 2014) have been conducted. For an example, in *C. gigas* PGRP, mRNA level was up-regulated after *Vibrio tubiashii* and *Marinococcus halophilus* challenge (Itoh and Takahashi, 2009). These data suggested that PGRPs from marine organisms play a crucial role in the innate immunity defense mechanisms against infections.

The *Bathymodiolus* mussel and Vesicomyidae clam are common species in hydrothermal vents and cold seeps (Barros et al., 2015). They have methane-oxidizing (MOX) bacteria or sulfur-oxidizing (SOX) bacteria in their gills as symbionts (Martins et al., 2014). Expression level of *BaPGRP* was up-regulated at 12 h but down-regulated at 24 h in the hydrothermal vents mussel *Bathymodiolus azoricus* that had been challenged with live *V. alginolyticus* (Martins et al., 2014). PGRPs from the cold seep mussel *B. platifrons* had different binding modes to peptidoglycan (PGN) from Gram-negative and Gram-positive microorganisms (Yue et al., 2015). These results suggested that pattern recognition receptors and related molecules are involved in the immune response of deep sea vent/seep organisms, but little information about Vesicomyidae clam is available.

The Vesicomyidae clam *A. packardana*, previously named Ca*lyptogena packardana*, which has been collected from cold seeps (Johnson et al., 2016). In present study, we study the roles of PGRP in immune system of this species. The primary objectives of the present research are: (1) identification and sequence analysis of ApPGRP-1 and -2 molecules; (2) combination and degradation of PGN by rApPGRP-1 and -2; (3) combination and inhibition of microbes by rApPGRP-1 and -2. The roles of PGRPs in the endosymbiosis relationship between the clam and endosymbiotic bacteria have also been discussed.

## Materials and Methods

### Sample Collection

Clams were collected from the Malibu Mound (33.902, -118.735) at a depth of 520 m during a MBARI expedition in 2014. Once clams were brought to the deck, the adductor muscle and gill were immediately removed and stored in RNAlater (Ambion, Austin, TX, United States). Total RNA was extracted and quality was assessed using gel electrophoresis and a spectrophotometer (Nanodrop2000, Thermo Scientific^TM^ NanoDrop^TM^, United States). Then, the RNA samples were sent out for sequencing at SeqMatic LLC (Fremont, CA, United States) on a HiSeq^TM^ 2000 platform. Over 6 Gbp clean data were obtained for each tissue library.

### Gene Identification and Expression Vector Construction

Homologs of the peptidoglycan recognition protein (PGRP) gene were found through searching the *A. packardana* transcriptome (data not yet published) using TBLASTN^1^ with previously published PGRP genes as a query. Two new PGRPs (ApPGRP-1 and -2) were identified.

cDNA was synthesized and amplified using a PrimeScript^TM^ II 1st Strand cDNA Synthesis Kit (Takara, Dalian, China) according to the manufacturer’s instructions. The open reading frame (ORF) of ApPGRP-1, -2 were amplified by PCR with primer pairs P1-1, P1-2 and P2-1, P2-2 (Table 1), and then the PCR products were inserted into plasmid expression vector pCOLDII(Takara, Dalian, China). The recombinant plasmids (pCOLDII-ApPGRP-1 and -2) were transformed into *Escherichia coli* Chaperone Competent Cells pG-KJE8/BL21 (Takara, Dalian, China). Colonies containing the appropriate vectors were verified by sequencing.

**Table 1:** Primers used in the present study.

Primer	Sequence (5′–3′)	Sequence information
P1-1 (sense)	ATGGAGCCACTTGTACA	ORF primer
P1-2 (antisense)	CTACCTTGTCATCTGATAC	ORF primer
P1-3 (sense)	CGGGATCCATGGAGCCACTT GTACA	Recombinant primer
P1-4 (antisense)	AACTGCAGCCTTGTCATCT GATAC	Recombinant primer
P2-1 (sense)	ATGCACGTGTATTCAATGT	ORF primer
P2-2 (antisense)	TTACGATGTTAGCTTATTCC CATCC	ORF primer
P2-3 (sense)	CGGGATCCAGGCACGTGGG CAGTTGTTCTGGCA	Recombinant primer (Signal peptide deleted)
P2-4 (antisense)	AACTGCAGCGATGTTAGCTT ATTCCCATCC	Recombinant primer

*Restriction enzyme target sites are underlined.*

### Sequence Analyses

Sequence comparison was conducted in the BLAST program^1^. We calculated theoretical isoelectric point and molecular weight in the ProtParam program^2^. SignalIP4.1^3^ was used to predict the signal peptide, and the SMART program (Letunic and Bork, 2018)^4^ was used to predict functional domains. Multiple protein sequences were aligned by using Clustal W program (Larkin et al., 2007) (version1.83^5^). A neighbor-joining (NJ) tree was established based on the deduced amino acid sequences with 1,000 bootstrap replicates by using MEGA v5.0 software (Tamura et al., 2011)^6^. The predicted tertiary structures were constructed in the SWISS-MODEL program^7^ and checked in Deepview/Swiss-Pdb Viewer 4.0^8^ (Guex and Peitsch, 1997).

### Expression, Purification and Western Blotting of Recombinant Proteins

Verified transformants were cultured in LB medium (yeast extract, 5 g, tryptone, 10 g, sodium chloride, 10 g of 1 L) with 100 μg/ml ampicillin, 20 μg/ml chloramphenicol, 0.5 mg/ml L-arabinose. The culture temperature was set at 37°C with shaking at 200 rpm. Two hours later, tetracycline was added into the LB medium to a final concentration of 2 ng/ml. L-arabinose and tetracycline are inducers of chaperone proteins dnaK-dnaJ-grpE and groES-groEL, respectively. Chloramphenicol is the resistance marker of plasmid pG-KJE8. When optical density at 600 nm (OD_600_) reached 0.5, the medium was cooled to 15°C and incubated for more than 30 min. After that, Isopropyl-h-d-thiogalactoside (IPTG) was added and the final concentration was 0.1 mM. The medium was cultured for another 24 h at 15°C. Then, bacteria were precipitated at 8,000 g for 5 min at 4°C. The recombinant proteins pCOLD II-ApPGRP-1 and -2 were present in the supernatant after sonication. Proteins were purified with Ni-NTA-Sefinose Column (Sangon Biotech, Shanghai, China), and eluted with 300 mM imidazole under non-denaturing conditions. The obtained proteins were dialysed, concentrated, and then stored at −80°C before use. The protein samples (before induction, after induction, the supernatant and precipitate after sonication) were separated in 10% SDS-polyacrylamide gel electrophoresis (SDS–PAGE). Western blotting procedures were set as follows: proteins were transferred from gels to PVDF membranes (Immobilon-membrane, Millipore, MA, United States) by Trans-Blot SD Semi-Dry Electrophoretic Transfer Cell (DYCP-40C, Beijingliuyi, Beijing, China). After that, the primary anti-6 × His antibody (diluted 1: 5000 with 5% skim milk; ab18184, abcam, Cambridge, United Kingdom) was incubated overnight with the membranes. The membrane was then washed with TBST (50 mM Tris–HCl, 50 mM NaCl, 0.05% Tween20, pH 7.2) three times. After that, secondary antibody (diluted 1: 10000; ab6789, abcam, Cambridge, United Kingdom) was incubated with the PVDF membranes for 2 h, followed by three washes with TBST. The membranes were incubated for 5 min with Pierce ECL Western Blotting Substrate (Thermo Scientific, MA, United States), and the Chemiluminescence imaging system (ChemStudio, Analytikjena, Jena, Germany) was used to detect chemical signals.

### Binding Analysis of rApPGRP-1, -2 to PGN

The assay was done as previous study (Yang et al., 2013) with slight modifications. We first incubated 40 μg of rApPGRP-1 or -2 proteins in 200 μl TBS buffer (50 mM Tris–HCl, 50 mM NaCl, pH 7.2) with L- PGN from *Staphylococcus. aureus* (Catalog No. 77140, Sigma-Aldrich, MA, United States; 100 μg, 1 mg/ml) and D-PGN from *Bacillus subtilis* (Sigma-Aldrich, MA, United States; 100 μg, 1 mg/ml). Then, bound and unbound proteins were obtained by centrifugation at 13,000 rpm for 15 min after incubation at 4°C for 3 h. TBS was used to wash the pellets (bound fraction) three times. After that, 2 × SDS–PAGE loading buffer was used to separate bound proteins from PGN by boiling at 95°C for 5 min. Samples were analyzed in 10% SDS–PAGE. Western blotting with anti-6 × His antibody was used to detect the target proteins as above.

### Binding Analysis of rApPGRP-1, -2 to Microbial Cells

The assay was done as described by Feng et al. (2012) with slight modifications. *S. aureus*, *M. luteus*, *B. subtilis*, *E. coli*, *Pichia pastoris*, and *Saccharomyces cerevisiae* colonies were grown in culture medium (LB medium for Gram-positive bacteria, Gram-negative bacteria; YPD medium for fungi). LB medium was the same as above, and YPD medium (1 L) includes: 10 g yeast extract, 20 g peptone, 20 g dextrose. When the OD_600_ was close to 0.8 (ca. 1.6 × 10^8^ cells/ml), 4 ml of each medium was centrifuged at 5,000 *g* and the pellets were washed twice with TBS buffer. Microbial cells were re-suspended in 50 μl of TBS buffer and then mixed with 40 μg of rApPGRP-1 or -2 dissolved in 200 μl of TBS buffer. The mixture was incubated for 3 h at 4°C, and centrifuged at 12,000 *g* at 4°C for 15 min. The cell pellets were washed three times with TBS buffer and then suspended in 50 μl of 2 × SDS sample buffer. The samples were heated at 95°C for 5 min to get the bound protein. The bound proteins from six kinds of microorganisms were checked in 10% SDS–PAGE and detected using western blotting as above.

### Analysis of Amidase Activities

Amidase activity of PGRP could cleave the lactylamide bond between muramic acid and L-alanine, and further cause the dissolution of the PGN (Foster, 2004). The assay was done as described by Mellroth et al. (2003), Yang et al. (2013) with slight modifications. 40 μg L, D-PGN (1 mg/ml) was incubated with 50 μg of rApPGRP-1 or -2 protein in TBS-ZnCl_2_ solution (50 mM Tris–HCl, 50 mM NaCl, 100 μM ZnCl_2_, pH 7.2). 40 μg PGN (1 mg/ml) was mixed with TBS buffer which was set as a control. OD_540_ was measured per 30 min during a 300 min period by using Varioskan LUX Multimode Microplate Reader (Thermo Fisher Scientific, MA, United States).

### Analysis of Antimicrobial Activities

The assay was done as described by Chen et al. (2014) with slight modification. *S. aureus*, *M. luteus*, *B. subtilis*, *E. coli*, *P. pastoris*, and *S. cerevisiae* were cultured to an OD_600_ of 0.6 in LB medium at 37°C or YPD medium at 28°C (Fungi). 200 μl aliquots of the cultures were pelleted by centrifugation at 5,000 *g* for 5 min at 4°C and washed with TBS buffer twice. The pellets were re-suspended in 200 μl TBS buffer. After a 1:50 dilution, 10 μl of the suspensions was mixed with 25 μl 200 μg/ml rApPGRP-1, or 200 μg/ml rApPGRP-2, or TBS (as control), with 10 or 100 μM ZnCl_2_ existed. The mixtures were shaken on a Tube Tumbler for 6 h at 26°C, and then 1 ml LB or YPD medium was added separately. The cultures were shaken at 37°C or 28°C overnight before absorbance measurement at 600 nm.

### Statistical Analyses

Statistical analysis was performed in SPSS22.0 (IBM Company, NY, United States). Duncan test of one-way ANOVA was used among mean values from different groups. The *P*-value is 0.05.

## Results

### Sequence Characteristics of ApPGRP-1 and -2

**FIGURE 1:**
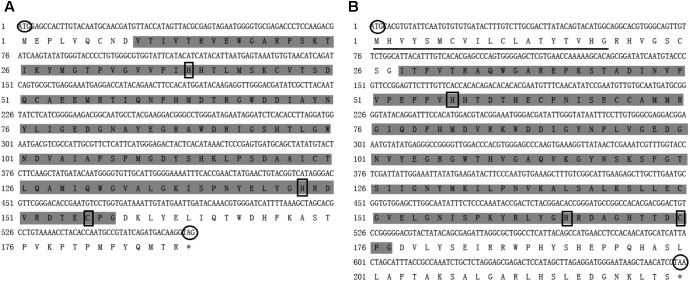
Nucleotide and deduced amino acid sequences of ApPGRP-1 **(A)** and ApPGRP-2 **(B)**. The predicted signal peptide is underlined, and the predicted start and termination codons are circled. The amidase/PGRP domain predicted in SMART program is highlighted in gray, and the black boxes indicate Zn^2+^ binding sites.

The open reading frame of ApPGRP-1 and ApPGRP-2 obtained using local blast are 567- and 675-bp, respectively (Genbank Nos. MH286797, MH306208, separately). ApPGRP-1 has 188 amino acids. Its theoretical isoelectric point and predicted molecular weight are 5.58, 21.38 kDa, respectively. ApPGRP-2 has 244 amino acids. Its theoretical isoelectric point and predicted molecular weight are 6.91, 24.94 kDa, respectively. ApPGRP-2 has a putative signal peptide in the N-terminus (Figure 1), but ApPGRP-2 doesn’t have. The Zn^2+^-dependent amidase domains are located in ApPGRP-1 and ApPGRP-2 at positions 10(V)–158(G) and 28(I)–177(G), respectively.

### Alignment and Phylogenetic Analysis of ApPGRP-1 and ApPGRP-2

Online BLAST analysis showed that amino acid sequences of ApPGRP-1 and -2 are highly homologous with PGRPs from other species. ApPGRP-1 is homologous with PGRPs from *H. cumingii* (AHK22786.1), *H. discus discus* (AHB30456.1), and the similarities are 58% and 53%, respectively. In addition, it also has a high homology with an *Octopus bimaculoides* protein (XP_014778613.1) with 56% identity. ApPGRP-2 has high similarity with PGRPs from *C. gigas* (XP_011422763.1) and *Pinctada fucata* (JAS03318.1), and the similarities are 56% and 52%, respectively. ApPGRP-1 and -2 have 44% similarity with each other.

**FIGURE 2:**
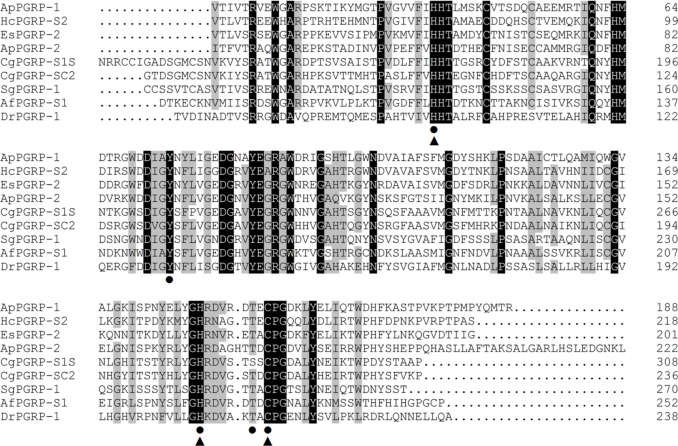
Alignments of amino acid sequences of ApPGRP-1 and -2 with other species. Zn^2+^ binding sites and amidase catalytic sites are marked with triangles 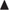 and cycles •, respectively. Amino acid residues that are conserved in at least 75% sequences are shaded in gray, and 100% identity amino acids are shaded in black. Species include CgPGRP-S1S (*Crassostrea gigas*, BAG31896), AfPGRP-S1 (*Azumapecten farreri*, AAY53765), SgPGRP-1 (*Solen grandis*, JN642118), EsPGRP-2 (*Euprymna scolopes*, AAY27974) and DrPGRP-1 (*Danio rerio*, NP_001037786), HcPGRP-S2 (*Hyriopsis cumingii*, AHK22786.1), and CgPGRP-SC2 (*C. gigas*, EKC26200.1).

Alignment analysis of these two ApPGRPs and other animals’ PGRPs showed that the C-portion of the PGRPs is highly similar and conserved, whereas the N-terminal region is relatively diversified (Figure 2). In ApPGRP-1, the Zn^2+^ binding sites (H39, H148, and C156) and amidase catalytic sites (H39, Y74, H148, T154, and C156) are both very conserved (Figure 2). The Zn^2+^ binding sites (H57, H166, and C175) and amidase catalytic sites (H57, Y92, H166, T173, and C175) are also conserved in ApPGRP-2 (Figure 2).

**FIGURE 3:**
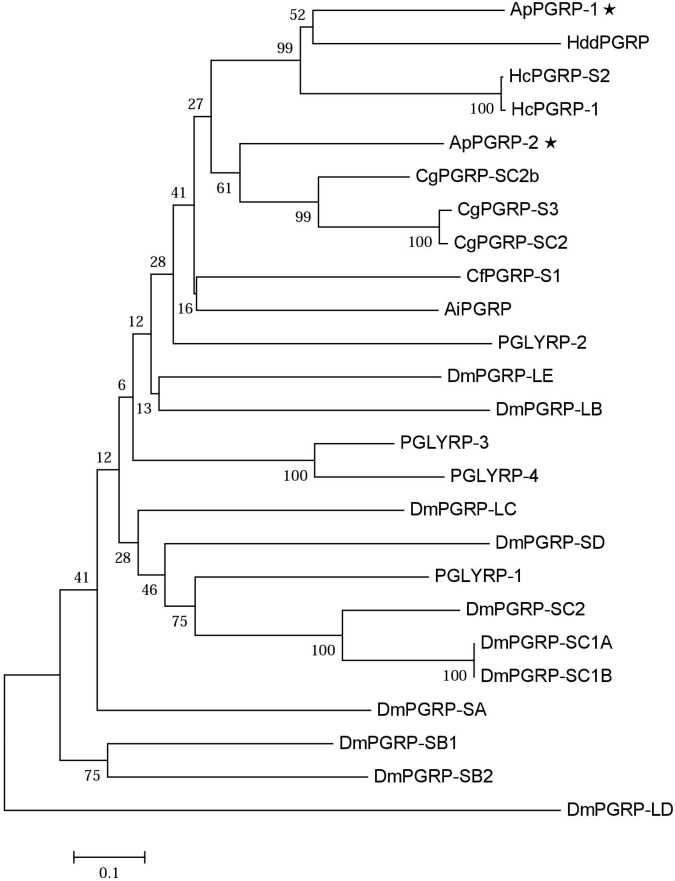
Phylogenetic tree of ApPGRP-1 and ApPGRP-2 with other PGRPs. CgPGRP-SC2 (XP_011422762.1), CgPGRP-SC2b (XP_011422763.1), and CgPGRP-S3 (BAG31899.1) from *C. gigas*, CfPGRP-S1 (AAY53765) from *Chlamys farreri*, AiPGRP (AAR92030) from *Argopecten irradians*, HcPGRP-1 (AGU68334.1) and HcPGRP-S2 (AHK22786.1) from *H. cumingi*, HddPGRP (AHB30456.1) from *Haliotis discus discus*, DmPGRP-LE (AAF48519), -SA (AAF48056), -LF (NP_648299), -LBa (AAF54643), -LCa (NP_729468), -LDa (NP_001027113), -SC1a (CAD89163), -SC1b (CAD89164), -SD (CAD89198), -SC2 (CAD89178), -SB1 (CAD89129), -SB2 (CAD89140), and -LAa (AAK00295) from *Drosophila melanogaster*, PGLYRP-1 (O75594), PGLYRP-2 (Q96PD5), PGLYRP-3 (Q96LB9), and PGLYRP-4 (Q96LB8) from *Homo sapiens*. The tree was established using the neighbor-joining (NJ) method using the Mega7.0 program based on coding sequences. Bootstrap values of 1,000 replicates (%) are indicated for the branches. ApPGRPs are labeled with ⋆.

Phylogenetic analysis of ApPGRP-1, -2 and other species’ PGRPs showed that ApPGRP-1 clustered with short PGRPs from abalone *H. discus discus* and freshwater pearl mussel *H. cumingii*, and ApPGRP-2 clustered with three short PGRPs from oyster *C. gigas*, and then these two branches were grouped together (Figure 3).

### Potential Tertiary Structures of ApPGRP-1 and -2

**FIGURE 4:**
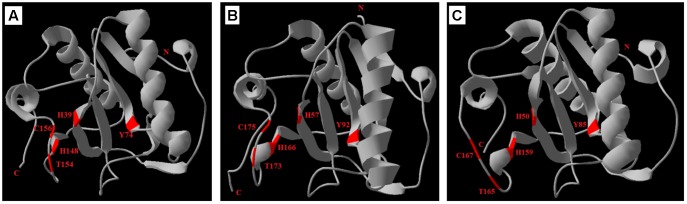
Predicted tertiary structures of ApPGRP-1, -2 and *Drosophila* PGRP-SC2 (CAD89187) using SWISS-MODEL. **(A,B)**: ApPGRP-1, -2; **(C)**: *Drosophila* PGRP-SC2. The Zn^2+^ binding sites and amidase catalytic sites are labeled in red.

The SWISS-MODEL prediction algorithm was used to predict the tertiary structures of ApPGRP-1, ApPGRP-2 and PGRP-SC2 from *Drosophila melanogaster* (CAD89187). The tertiary and secondary protein structure of ApPGRP-1 and -2 are well conserved by comparing with model *Drosophila* PGRP-SC2 (Figure 4). ApPGRP-1 was predicted to have four α-helices and seven β-strands (refer to the whole protein sequences). ApPGRP-2 was predicted to have five α-helices and eight β-strands (refer to the whole protein sequences). PGRP from *D. melanogaster* has a typical PGRP structure including five β-strands and five α-helices. The Zn^2+^ binding sites and amidase catalytic sites in ApPGRP-1 and-2 are both well conserved (Figure 2), while H50-H159-C167 and H50-Y85-H159-T165-C167 are present in PGRP-SC2 from *D. melanogaster* (Figure 4).

### Bioassay of Recombinant ApPGRP-1 and -2 Proteins

**FIGURE 5:**
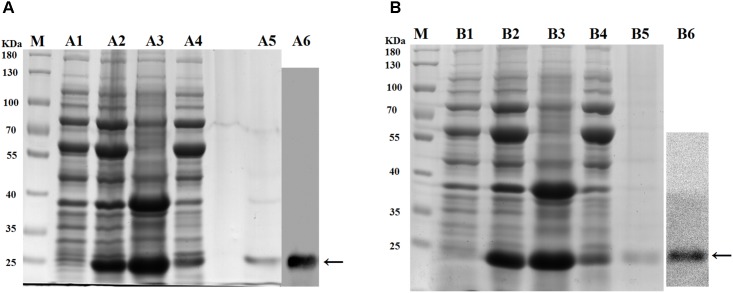
SDS–PAGE of recombinant proteins and western blotting. **(A)**: rApPGRP-1, **(B)**: rApPGRP-2. lane M, molecular mass standards; lane A1, B1, total cellular extracts from *Escherichia. coli* Chaperone Competent Cells pG-KJE8/BL21 with pCold II before induction; lanes A2, B2, total cellular extracts from IPTG induced *E. coli* cells (containing expression vector); lanes A3, B3, pellet of total cellular extracts from IPTG induced *E. coli* (containing expression vector); lanes A4, B4, supernatant of total cellular extracts from IPTG induced *E. coli* (containing expression vector); lanes A5, B5, purified rApPGRP-1 and rApPGRP-2; lanes A6, B6, western blot. The bands of target protein are labled by ←.

A clear band with a molecular mass of ∼24 kDa (containing partial vector sequences; Figure 5A-A5) was detected (→ label), which match predicted protein size of rApPGRP-1 (Figures 5A-A1–A4). The western blotting results in Figure 5A-A6. Lane B5 in Figure 5B showed a unique ∼28 kDa protein representing rApPGRP-2.

### Binding of Recombinant Protein ApPGRP-1 and -2 to L, D-PGN

**FIGURE 6:**
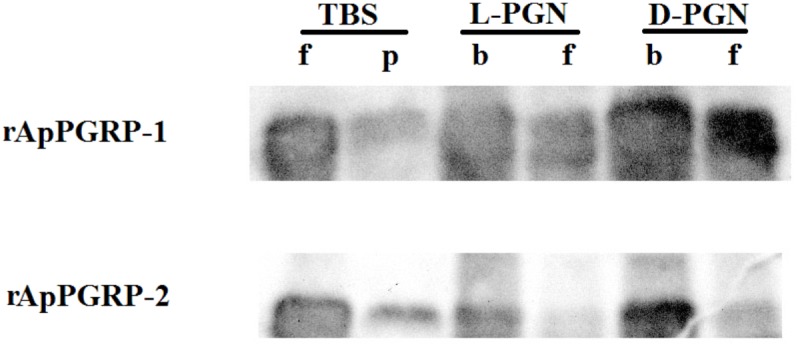
rApPGRP-1 and -2 bind Lys-type PGN from *S. aureus* and Dap-type PGN from *B. subtilis*. f, free; p, pellet; b, bound.

Binding of rApPGRP-1 and -2 to PGN (L-PGN and D-PGN) was analyzed using western blotting. For the TBS group in Figure 6, most of the rApPGRP-1 and -2 protein was present in the supernatant. For the L-PGN group, rApPGRP-1 bound it, although there was also an obvious band corresponding to the unbound fraction. Binding of rApPGRP-2 to L-PGN was relatively strong as there was no clear band in the unbound part. For the D-PGN group, a similar pattern was existed (Figure 6). Both rApPGRP-1 and -2 could bind L- PGN or D-PGN.

### Binding of Recombinant ApPGRP-1 and -2 Proteins to Microbial Cells

**FIGURE 7:**
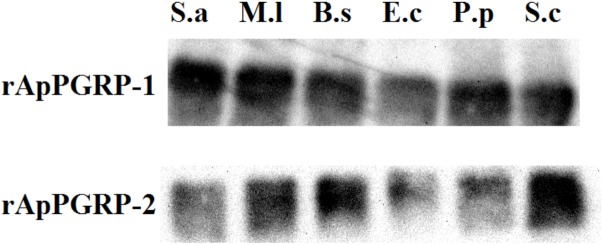
The combination of rApPGRP-1 and -2 with live microbes as detected using western blot. S.a, *Staphylococcus aureus*; M.l, *Micrococcus lutea*; B.s, *Bacillus subtilis*; E.c, *Escherichia coli*; P.p, *Pichia pastoris*; S.c, *Saccharomyces cerevisiae.*

Microbe binding assay was done to analyze whether rApPGRP-1 and -2 bound Gram-negative bacteria, Gram-positive bacteria and fungi. Clear bands were detected which suggested that rApPGRP-1 and -2 could bind to six microbes (Figure 7). The band intensities of E.c (lane 4, Figure 7) were weaker than other bands. No bands were observed for the negative control (data not shown). Seeing from these data, the ApPGRPs proteins could bind a wide spectrum of bacteria.

### Amidase Activities of rApPGRP-1 and -2

**FIGURE 8:**
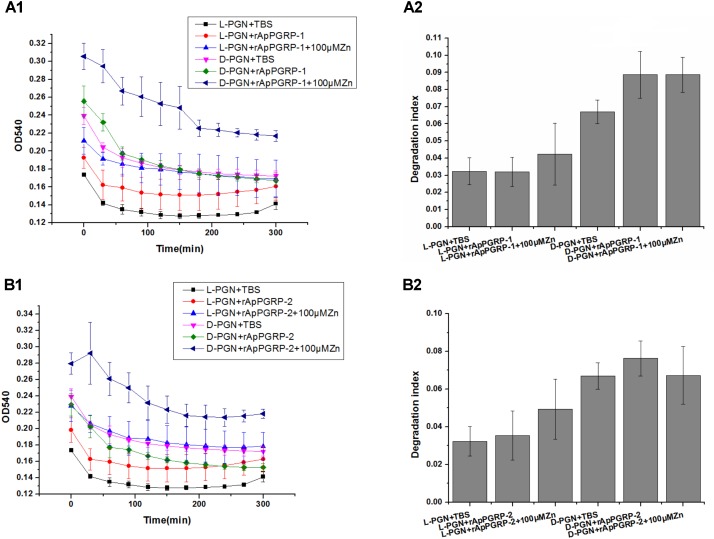
Activity of rApPGRP-1 and -2 in the degradation of L-PGN and D-PGN. **(A1,A2)**, OD_540_ change and degradation index of rApPGRP-1, respectively; **(B1,B2)**, OD_540_ change and degradation index of rApPGRP-2, respectively. Degradation index = OD_540_(0 min)–OD_540_(300 min).

With L- and D-PGN as substrates, we estimated relative amidase activities of rApPGRP-1 and -2 through measuring OD_540_ value. The OD_540_ value went down dramatically within 300 min when the TBS group was incubated with L-PGN or D-PGN (Figure 8). rApPGR-1 and -2 degraded L-PGN in the existence of Zn^2+^ (except D-PGN + rApPGRP-1 + 100 μMZn group and D-PGN + rApPGRP-2 + 100 μMZn group). rApPGRP-1 degraded D-PGN in the absence of Zn^2+^ (ANOVA, *F*(1,5) = 6.036, *P* = 0.07; Figure 8A2). Zn^2+^ did not enhance this amidase activity as no change was found between D-PGN + rApPGRP-1 group and D-PGN + rApPGRP-1 + 100 μMZn group. A similar result could be seen in rApPGRP-2 (Figure 8B2), whereas Zn^2+^ might slightly inhibit rApPGRP-2 from degrading D-PGN. As there are conserved catalytic residues in rApPGRP-1 and -2, we concluded that rApPGRP-1 and -2 have amidase activity against both Lys-type and Dap-type PGN (Figure 8).

### Antimicrobial Activities of rApPGRP-1 and -2

**FIGURE 9:**
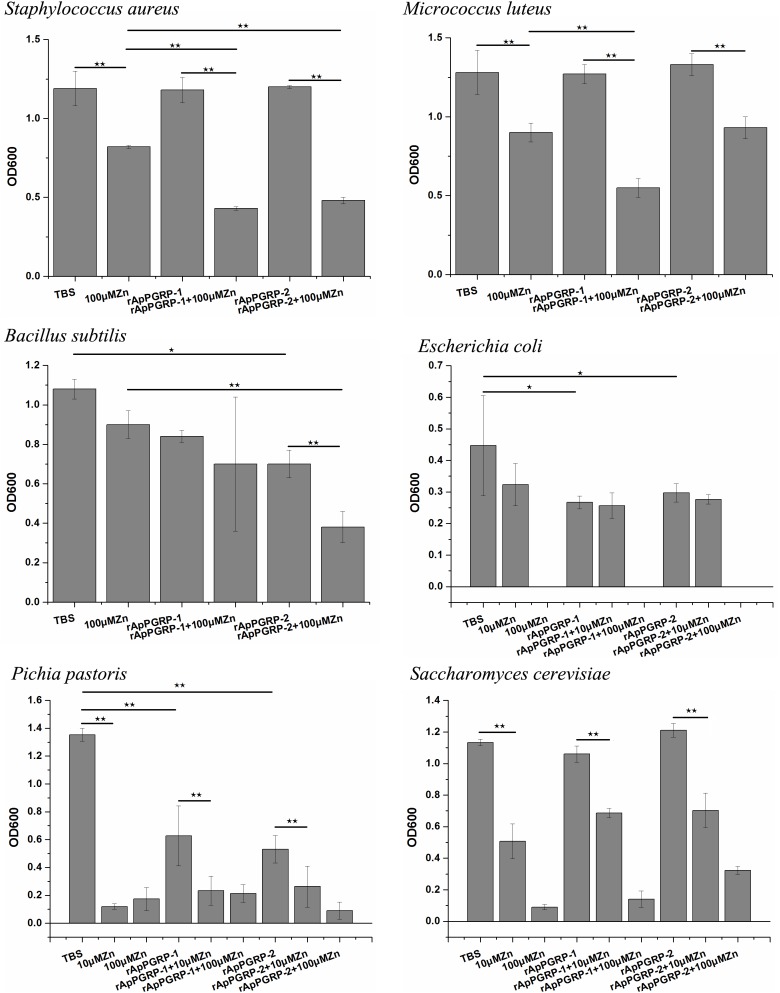
Antimicrobial activity of rApPGRP-1 and -2. ⋆⋆ indicates *P* < 0.01, ⋆ indicates *P* < 0.05.

For Gram-positive bacteria, when zinc ions didn’t exist, rApPGRP-2 had antibacterial activity against *B. subtilis*, but no antibacterial activity against *S. aureus* or *M. luteus*. In the participation of zinc ions, rApPGRP-1 + 100 μMZn group significantly inhibited *S. aureus*, which was significant different from the rApPGRP-1 or 100 μMZn groups (*P* < 0.01), indicating that rApPGRP-1 had strong enough amidase activity to achieve bactericidal activity in the presence of zinc ions. Similarly, rApPGRP-2 + 100 μMZn group showed analogous activity against *B. subtilis* and rApPGRP-1 + 100 μMZn group showed activity against *M. luteus* (Figure 9).

For *E. coli*, *S. cerevisiae* and *P. pastoris*, we chose 10 μM Zn^2+^ for antibacterial experiments, as 100 μM Zn^2+^ alone had a strong antibacterial effect on these microorganisms.

For *E. coli*, rApPGRP-1 and -2 had clear antimicrobial activity in the absence of 10 μM Zn^2+^. In the presence of 10 μM Zn^2+^, 10 μMZn group had a bacteriostatic effect but not significant. As the antibacterial effect of rApPGRP-1 + 10 μMZn group is not more than rApPGRP-1 group and there is no significant difference among these three groups: rApPGRP-1, 10 μMZn and rApPGRP-1 + 10 μMZn group (*P* > 0.05), suggested that antibacterial ability of rApPGRP-1 may not require zinc ions. A similar situation was also observed in the rApPGRP-2 + 10 μMZn group (Figure 9).

For fungi *S. cerevisiae*, rApPGRP-1 and -2 had no antibacterial activity in the absence of 10 μM Zn^2+^. In the presence of 10 μM Zn^2+,^ the antibacterial activity of 10 μMZn, rApPGRP-1 + 10 μMZn and rApPGRP-2 + 10 μMZn groups were not significantly different from each other (*P* > 0.05), indicating that the antibacterial ability of rApPGRP-1 + 10 μMZn and rApPGRP-2 + 10 μMZn groups may derive from the zinc ions. For *P. pastoris*, a similar pattern to *S. cerevisiae* was also observed for rApPGRP-1 and-2 when 10 μM Zn^2+^ existed, but both proteins had significant antimicrobial activity in the absence of 10 μM Zn^2+^ (*P* < 0.01; Figure 9).

From these analyses, rApPGRP-1 and -2 showed zinc-dependent or -independent bactericidal activity.

## Discussion

In this study, two new short PGRPs (ApPGRP-1 and -2) were identified from *A. packardana*. Homology analysis indicated that ApPGRPs had relatively high similarity with PGRPs of other organisms, and phylogenetic tree analysis showed that ApPGRPs clustered with most PGRPs from molluscs with high bootstrap values. As mollusc PGRPs are commonly involved in a series of immune responses, rApPGRP-1 and -2 from *A. packardana* might also play a similar role in regulating diverse immune responses to adapt cold seep habitat.

### PGN Binding Specificity and Amidase Activities of PGRPs

L-PGN or D-PGN can be specifically and preferentially recognized by PGRPs. In *Drosophila*, L-PGN from Gram-positive bacteria could trigger the toll signal pathway by PGRP-SA or PGRP-SD. Dap-type PGNs from *Bacillus* and Gram-negative bacteria could stimulate the IMD pathway (Michel et al., 2001; Hoffmann, 2003; Kaneko et al., 2004). In molluscs, rCfPGRPS1 from *Chlamys farreri* displays affinity to L-PGN from *S. aureus* (Yang et al., 2010). CgPGRP-S1S from pacific oyster displays specific binding activity to D-PGN, but not to L-PGN (Iizuka et al., 2014). In this study, PGN binding assays revealed that ApPGRPs have PGN-binding activity toward D, L-PGN which was also found in HcPGRPS1 from *H. cumingi* (Yang et al., 2013). It could be seen that PGRPs from different organisms have their specific PGN binding spectrums.

We also measured amidase activities of the PGRPs. In rPGRP-S from amphioxus, with the participation of Zn^2+^, higher hydrolyzing activity was detected when using Lys-PGN as substrate compared to Dap-type PGN (Yao et al., 2012). In the present study, rApPGRP-1 and -2 showed some but not significant degradation activity for L-PGN in the presence of Zn^2+^. However, rApPGRP-1 also showed somehow amidase activity toward D-PGN in the absence of Zn^2+^. These results are consistent with previous study (Chen et al., 2014). We therefore conclude that the amidase activity of these ApPGRPs is Zn^2+^-selective dependent.

### Microbial Binding Specificity and Antibacterial Activity

rApPGRP-1 and -2 bound six bacteria with a broad spectrum. Recombinant ApPGRP-1 exhibited antibacterial activity that inhibited the growth of *S. aureus* and *M. luteus* in the presence of Zn^2+.^ This is consistent with the results from *Drosophila* (Mellroth and Steiner, 2006), *C. farreri* (Yang et al., 2010). On the other hand, rApPGRP-1 and-2 had obvious antimicrobial activity for *E. coli* and *P*. *pastoris* in the absence of Zn^2+^. This antimicrobial activity might similar to BmPGRP-S5 from *Bombyx mori*, which has obvious antibacterial activity toward Gram-positive bacteria *M. luteus* and *S. aureus*, Gram-negative bacteria *S. marcescens*, and *E. coli* (Chen et al., 2014). Similarly, amphioxus rPGRP-S can also suppress the growth of *P. pastoris* in the absence of Zn^2+^ (Yao et al., 2012). In our study, rApPGRP-1 and -2 displayed amidase activity and bactericidal activity in Zn^2+^-dependent or –independent manner. These results indicated that the bactericidal effect of ApPGRPs might be a direct effect of amidase-mediated lysis of PGN as there are conserved sites (H-Y-H-T-C) for amidase activity in ApPGRP-1, -2.

### Functions of ApPGRPs

**FIGURE 10:**
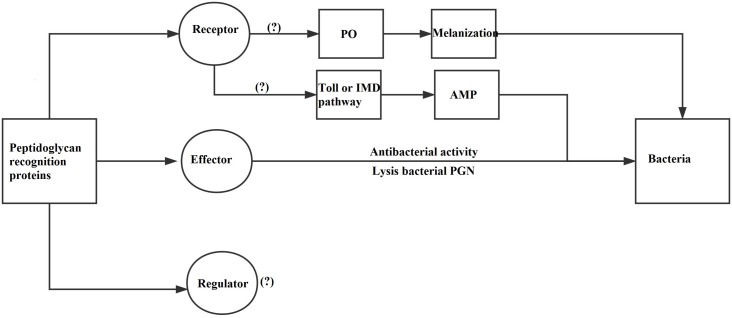
A diagram of PGRPs’ functions [modified from Chen and Lv (2014)]. PO, Phenoloxidase; IMD, Immune Deficiency; AMP, Antimicrobial peptide.

Peptidoglycan recognition proteins mainly have three functions: recognition receptor, regulator, and effector (Figure 10; Chen and Lv, 2014). ApPGRPs bound PGN and six kinds of microorganisms, which indicate they have partial functions of PGRPs as receptors. After that, IMD, PO, or Toll pathway would be activated, and hosts produce diverse immune responses (Michel et al., 2001; Bischoff et al., 2004; Park et al., 2007; Pal and Wu, 2009). However, the roles of ApPGRPs in these pathways are still not well studied. From the result that ApPGRPs have amidase activity to degrade PGN and could kill microorganisms, we speculate that they can function as effector of PGRPs. To explore the possible roles of ApPGRPs as a regulator, further *in vivo* study is needed.

### Trade Off Between Immune Response and Symbiotic Relationship Maintaining

The clam *A. packardana* in the study is from cold seep and has sulfur-oxidizing in its gills as a symbiont (Johnson et al., 2016). ApPGRPs can bind microbes, cleave PGN and display antibacterial activity, which indicated that PGRPs from *A. packardana* might play vital roles in regulating diverse immune responses. Organisms that incorporate symbiotic bacteria into their bodies must maintain a stable symbiotic relationship, therefore as a regulator, PGRPs might play important roles in this process. Given that the large amount symbionts are existed in cold seep clam gills (Johnson et al., 2016), ApPGRPs might function as a regulator (enhance or inhibit) in this tissue. To maintain a long-term symbiotic relationship, weevils from the genus *Sitophilus* express a PGRP which can decrease the biological activity of PGN from symbiotic bacteria (maybe), and therefore avoid PGN from stimulating the host to generate higher immune response (Anselme et al., 2006; Moya et al., 2008). In deep sea organisms, PGRPs’ roles as regulator are not very clear. We just found that PGRPs from the Atlantic vent mussel *B. azoricus* might participate in the immune response at early point as they establish symbiotic relationship with bacteria (Martins et al., 2014). PGRPs from cold seep mussel *B. platifrons* might have different roles in different tissues, and BpPGRPs might recognize symbiont bacteria in the gill and function in the immune response in the visceral mass (Sun et al., 2017).

## Conclusion

In conclusion, two short PGRPs from the cold seep clam *A. packardana* were identified and preliminarily analyzed. Functional assays showed that ApPGRPs have biological function and participate in the immune response. By comparison with PGRPs from other invertebrates, we hypothesize that ApPGRP might also be involved in the endosymbiosis relationship between the host and endosymbiotic bacteria as a regulator. Taken together, our study provides some basic information for further study on the immune and symbiotic mechanisms of vent/seep molluscs.

## Ethics Statement

All applicable international, national, and/or institutional guidelines for the care and use of animals were followed.

## Author Contributions

XK and HZ conceived and designed the experiments, analyzed the data, and wrote the paper. XK, HL, YL, and HZ performed the experiments. All authors reviewed the manuscript.

## Conflict of Interest Statement

The authors declare that the research was conducted in the absence of any commercial or financial relationships that could be construed as a potential conflict of interest.

## Footnotes

1 http://www.ncbi.nlm.nih.gov/BLAST/

2 http://www.expasy.ch/tools/protparam.html

3 http://www.cbs.dtu.dk/services/SignalP/

4 http://smart.embl-heidelberg.de/

5 http://www.clustal.org/clustal2/

6 http://www.megasoftware.net/mega4/

7 http://swissmodel.expasy.org/

8 https://spdbv.vital-it.ch/

## Abbreviations

PAMPs: pathogen associated molecular patterns
PRRs: pattern-recognition receptors
PGRPs: Peptidoglycan recognition proteins
PO: Phenoloxidase
IMD: Immune Deficiency
AMP: Antimicrobial peptide.
